# Establishment and evaluation of a risk-prediction model for hypertension in elderly patients with NAFLD from a health management perspective

**DOI:** 10.1038/s41598-022-18718-3

**Published:** 2022-09-07

**Authors:** An Zhang, Xin Luo, Hong Pan, Xinxin Shen, Baocheng Liu, Dong Li, Jijia Sun

**Affiliations:** 1 Department of Health Management, School of Public Health, Shanghai University of Traditional Chinese Medicine, Shanghai, 201203 China; 2 Shanghai Collaborative Innovation Center of Traditional Chinese Medicine Health Service, Shanghai University of Traditional Chinese Medicine, Shanghai, 201203 China; 3 Zhangjiang Community Health Service Centers, Pudong New Area, Shanghai, 201203 China

**Keywords:** Computer modelling, Systems biology, Risk factors, Machine learning, Predictive medicine

## Abstract

Elderly patients with nonalcoholic fatty liver disease are at a higher risk of developing. This study established an effective, individualised, early Hypertension risk-prediction model and proposed health management advice for patients over 60 years of age with NAFLD. Questionnaire surveys, physical examinations, and biochemical tests were conducted in 11,136 participants. The prevalence of NAFLD among 11,136 participants was 52.1%. Risk factors were screened using the least absolute shrinkage and selection operator model and random forest model. A risk-prediction model was established using logistic regression analysis and a dynamic nomogram was drawn. The model was evaluated for discrimination, calibration, and clinical applicability using receiver operating characteristic curves, calibration curves, decision curve analysis, net reclassification index (NRI), and external validation. The results suggested that the model showed moderate predictive ability. The area under curve (AUC) of internal validation was 0.707 (95% CI: 0.688–0.727) and the AUC of external validation was 0.688 (95% CI: 0.672–0.705). The calibration plots showed good calibration, the risk threshold of the decision curve was 30–56%, and the NRI value was 0.109. This Hypertension risk factor model may be used in clinical practice to predict the Hypertension risk in NAFLD patients.

## Introduction

Nonalcoholic fatty liver disease (NAFLD) is an acquired metabolic disease characterised by fatty deposits in the liver^1^. The prevalence of NAFLD is increasing with the standard of living of the population^2^, and it is gradually becoming the most common chronic liver disease worldwide, posing an increasing economic and medical burden^3,4^. NAFLD prevalence is higher in China than in some developed countries because of the variability of disease prevalence and lifestyle changes. A projection of the future burden of NAFLD shows China is expected to have 314 million patients with NAFLD by 2030^5^. Some studies have shown that NAFLD patients have a higher risk of cardiovascular diseases, such as hypertension, coronary heart disease, cardiomyopathy, and cardiac arrhythmias, than the general population^6,7^.

Hypertension is a common cardiovascular condition responsible for 9.4 million deaths worldwide every year^8^. Its clinical diagnostic criteria are a systolic blood pressure (SBP) ≥ 140 mmHg or diastolic blood pressure (DBP) ≥ 90 mm Hg^9^. China ranks first worldwide for deaths due to Hypertension, and the proportion elderly people suffering from Hypertension in China is > 60%^10,11^. Recent studies have found that the risk of hypertension is notably higher in patients with NAFLD than in other populations^12–14^, and there is a strong association between the two. An observational study showed that NAFLD was associated with a 1.6-fold risk of developing hypertension^15^. The prevalence of NAFLD combined with hypertension is approximately 39.34%^2,16,17^, and the quality of life of patients with NAFLD-Hypertension is lower than for those with either condition alone.

Clinical prediction models (CPMs) are used as a quantitative tool for assessing the risks and benefits of particular clinical scenarios. They can provide medical practitioners with scientifically valid data and their application is becoming increasingly common^18^. However, most CPMs are established in developed countries, with only a few being applied in developing and underdeveloped countries. Through a literature review, we found few reports on the establishment of the CPMs for Hypertension in China, and reports differed significantly between regions^8,19–22^. Therefore, it would be of great practical value to establish CPMs for different regions and populations in China and propose health management strategies based on the research findings.

The Hypertension risk-prediction model for elderly patients with NAFLD developed in this study could, to some extent, fill the research gap in terms of regions and populations. Using multiple indicators obtained from questionnaires, physical examinations and biochemical tests, we can predict the risk of concurrent Hypertension with NAFLD in elderly patients. This can therefore help medical practitioners to identify and select high-risk individuals for non-medical health interventions at an early stage to delay or prevent NAFLD patients from developing Hypertension.

## Results

### Basic statistical description

The basic demographic information and clinical examination results of the study population were collected primarily through questionnaires, physical examinations, and biochemical tests. After processing, the final 24 basic variables in the analysis were: age, SBP, DBP, body mass index (BMI), waist-to-hip ratio (WHR), and the concentrations in each sample of albumin (ALB), alanine aminotransferase (ALT), aspartate aminotransferase (AST), red blood cells (RBC), UREA, glucose (GLU), haemoglobin (HGB), platelets (PLT), total cholesterol (TC), total bilirubin (TB), creatinine (CRE), low-density lipoprotein (LDL) triglycerides (TG), uric acid (UA), alpha-fetoprotein (AFP), basophils (BASO), eosinophils (EOS), lymphocytes (LYMPH), and neutrophils (NEUT).

The data analyses of the 5287 samples collected in this study in 2017 are shown in Table 1, illustrating the basic statistical description of the sample data for NAFLD and non-NAFLD groups. The NAFLD group is used as an internal training set. The 24 variables used in the analysis do not meet the normal distribution assumption due to the large sample size. The mean ± standard deviation was used to describe the results, and the chi-squared test and *t*-test were used to analyse whether the differences between the groups were statistically significant.

**Table 1:** Differences in demographic and clinical characteristics between the No-NAFLD and NAFLD groups in training set. [mean ± SD or N (%)].

Demographic characteristics	NAFLD (n = 2845)	No-NAFLD (n = 2442)	$\chi^{2} /t$	P
**Hypertension**				
Yes	1714 (60.2%)	1124 (46.0%)	106.838	< 0.001
No	1131 (39.8%)	1318 (54.0%)		
Age (years)	69.01 ± 6.92	69.39 ± 7.36	1.897	0.058*
BMI (kg/m^2^)	25.37 ± 2.87	22.57 ± 3.11	33.730	< 0.001
SBP (mmHg)	144.59 ± 21.24	139.33 ± 22.80	8.635	< 0.001
DBP (mmHg)	82.26 ± 11.35	80.64 ± 12.10	5.017	< 0.001
WHR	0.90 ± 0.06	0.86 ± 0.06	20.773	< 0.001
ALB (g/L)	44.53 ± 2.53	43.96 ± 2.71	7.843	< 0.001
ALT (U/L)	25.97 ± 13.94	21.48 ± 11.03	13.071	< 0.001
AST (U/L)	23.74 ± 8.06	23.04 ± 6.68	3.467	0.001
RBC (10^12^/L)	4.63 ± 0.46	4.51 ± 0.48	9.329	< 0.001
UREA (mmol/L)	5.51 ± 1.50	5.43 ± 1.53	1.950	0.051*
GLU (mmol/L)	6.38 ± 1.80	5.92 ± 1.58	9.826	< 0.001
HGB (g/L)	141.39 ± 13.60	138.68 ± 14.44	6.994	< 0.001
PLT (10^9^/L)	200.69 ± 54.66	192.28 ± 57.19	5.438	< 0.001
TC (mmol/L)	5.11 ± 0.96	5.05 ± 0.98	2.111	0.035
TB (μmol/L)	15.71 ± 5.79	15.81 ± 5.62	0.642	0.521*
CRE (μmol/L)	68.76 ± 17.74	70.51 ± 18.28	3.526	< 0.001
LDL (mmol/L)	3.21 ± 0.86	3.13 ± 0.86	3.634	< 0.001
TG (mmol/L)	1.71 ± 1.21	1.21 ± 9.75	18.529	< 0.001
UA (μmol/L)	358.71 ± 87.56	327.51 ± 82.15	13.287	< 0.001
AFP (ng/mL)	6.88 ± 4.67	6.89 ± 5.60	0.056	0.955*
BASO (10^9^/L)	0.02 ± 0.03	0.03 ± 0.05	2.411	0.016
EO (10^9^/L)	0.14 ± 0.14	0.13 ± 0.12	2.261	0.024
LYMPH (10^9^/L)	1.95 ± 0.59	1.79 ± 1.31	5.679	< 0.001
NEUT (10^9^/L)	3.40 ± 1.14	3.17 ± 1.10	7.378	< 0.001

"*" means *p* > 0.05, and the difference was not statistically significant.

### Screening results of characteristic variables

**Figure 1:**
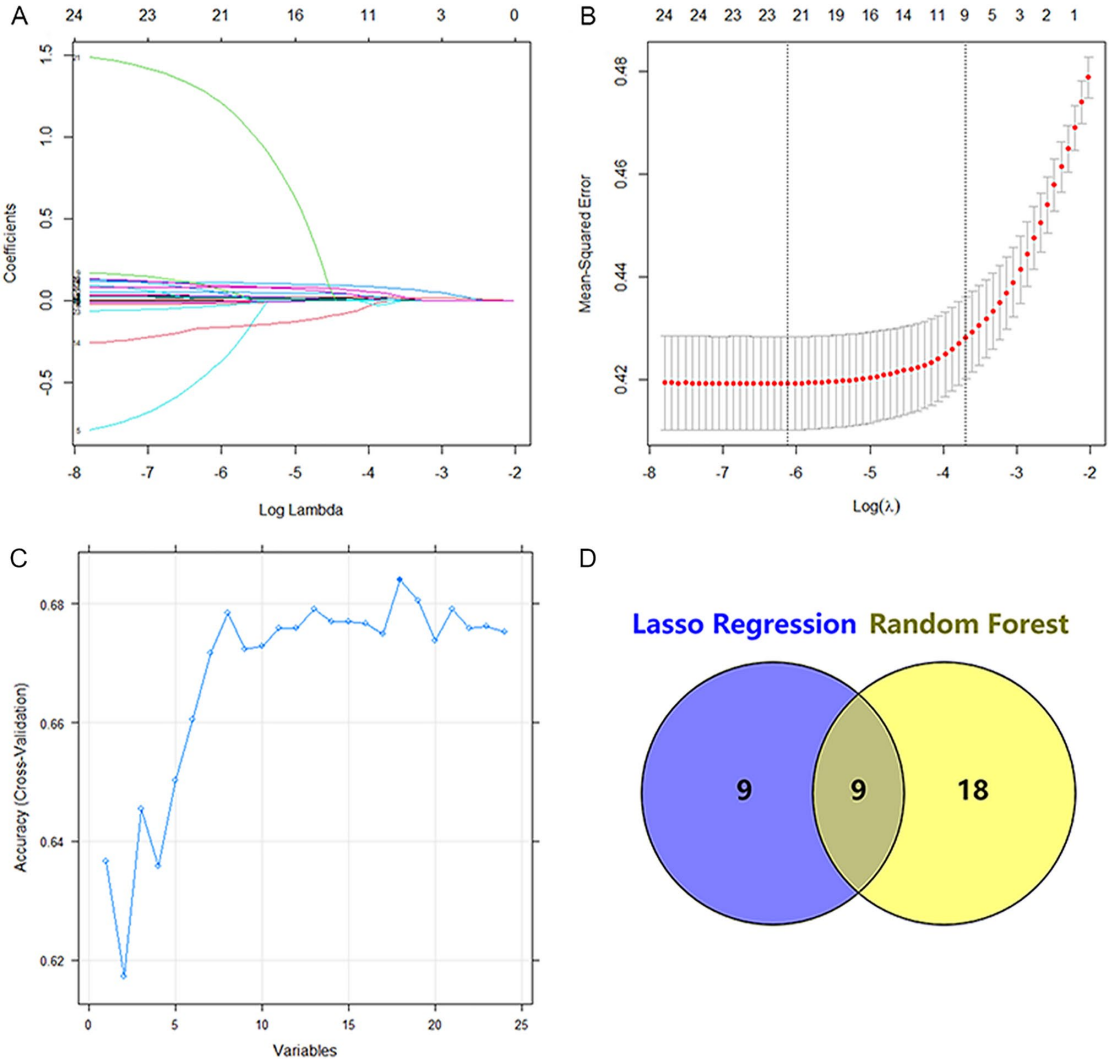
Variable screening using the Lasso regression model. (**A**) Nine variables with non-zero coefficients were selected by deriving the optimal lambda values. (**B**) Coefficient profiles are generated according to optimal parameters (lambda) in (**A**). (**C**) Resulting graph of variable selection using the random forest model method. (**D**) Schematic diagram of the method for selection of the final characteristic variables in this study.

**Figure 2:**
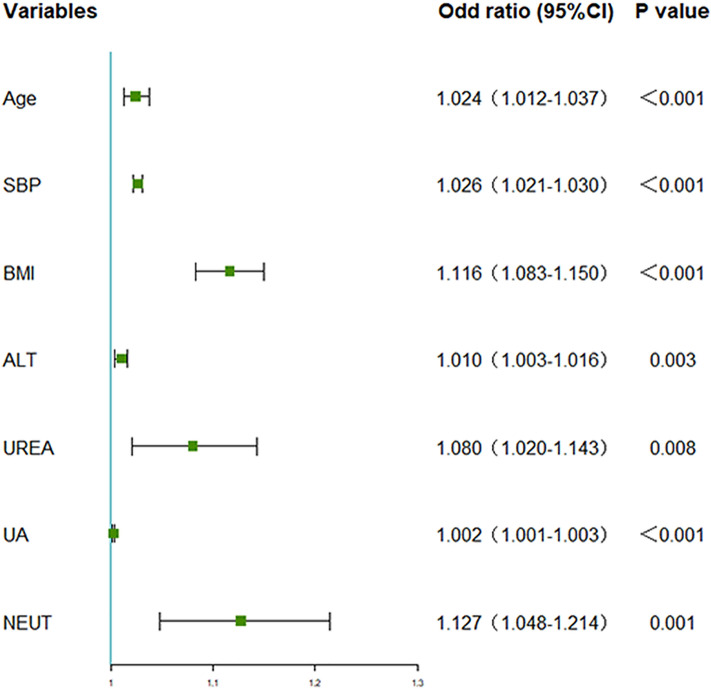
Forest plot of the selected elements. A forest plot is used to visualise the results of the logistic regression analysis.

In the training set, nine characteristic variables out of 24 basic variables were screened for using the Lasso model and intersected with 18 characteristic variables screened for using RF to obtain the following common variables: age, SBP, BMI, ALT, UREA, TC, LDL, UA, and NEUT (Fig. 1). These nine common variables were included in the multivariate logistic regression analysis and as a result of which the variables of age, SBP, BMI, ALT, UREA, UA, and NEUT were maintained as the final selection after excluding the variables with *p* > 0.05 (Fig. 2, Table 2).

**Table 2:** Predictive factors for hypertension incidence risk in NAFLD patients.

Intercept and variable	Prediction model					
	β	Z value	*P* value	OR	2.5% CI	97.5% CI
Intercept	− 9.534	− 14.86	< 0.001	7.237e^− 05^	2.029e^−05^	2.512e^−04^
Age	0.024	3.79	< 0.001	1.024	1.012	1.037
SBP	0.025	11.80	< 0.001	1.026	1.021	1.030
BMI	0.109	7.12	< 0.001	1.116	1.083	1.150
ALT	0.010	2.95	0.003	1.010	1.003	1.016
UREA	0.077	2.63	0.008	1.080	1.020	1.143
UA	0.002	4.78	< 0.001	1.002	1.001	1.003
NEUT	0.120	3.20	0.001	1.127	1.048	1.214

### Model construction and prediction results

Using the seven indicators selected above as independent variables and "whether NAFLD patients have concurrent Hypertension" as the dependent variable, a model was constructed using logistic regression to obtain a prediction model for the risk of concurrent Hypertension in elderly NAFLD patients:

$$\begin{aligned} y_{{{\text{model}}}} = & - 9.534 + 0.024 \cdot {\text{Age}} + 0.025 \cdot {\text{SBP}} + 0.109 \cdot {\text{BMI}} \\ & + 0.010 \cdot {\text{ALT}} + 0.077 \cdot {\text{UREA}} + 0.002 \cdot {\text{UA}} + 0.120 \cdot {\text{NEUT}} \\ \end{aligned}$$

**Figure 3:**
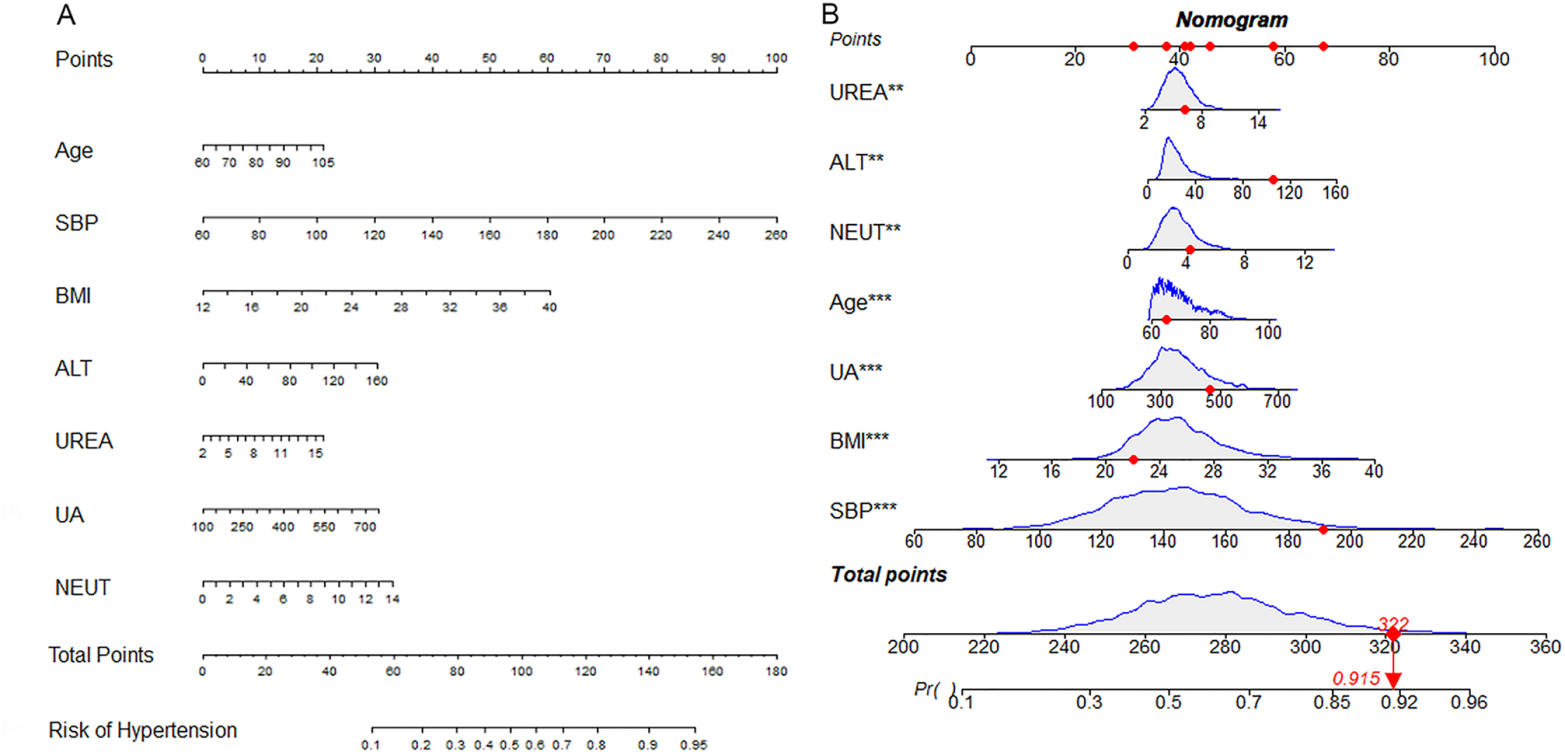
The risk nomogram of Hypertension in patients with NAFLD. (**A**) Nomogram including age, SBP, BMI, ALT, UREA, TC, LDL, UA, and NEUT for analysis. (**B**) One patient with NAFLD was randomly selected, whose Hypertension incidence was predicted based on the seven characteristic indicators of the nomogram.

The Hypertension risk in patients with NAFLD can be simply and visually predicted based on the nomogram drawn from this model (Fig. 3). For example, suppose a patient with NAFLD had the following characteristics: age 65 years, BMI 25.5 kg/m^2^, SBP 191 mmHg, ALT 106 U/L, UREA 6.2 mmol/L, UA 465.9 μmol/L, and NEUT 4.2⋅10^9^/L. This produces a score of 322, corresponding to a probability of 0.915, indicating that the risk of concurrent Hypertension in this NAFLD patient is 91.5%. In addition, we created an online app to predict the probability of concurrent Hypertension in elderly patients with NAFLD based on the model established in this study. The website is https://studentluo.shinyapps.io/DynNomapp/. This app can help clinicians diagnose Hypertension and community medical practitioners design reasonable and effective primary and secondary prevention management programs for Hypertension with NAFLD in elderly patients.

### Model evaluation and comparison

**Figure 4:**
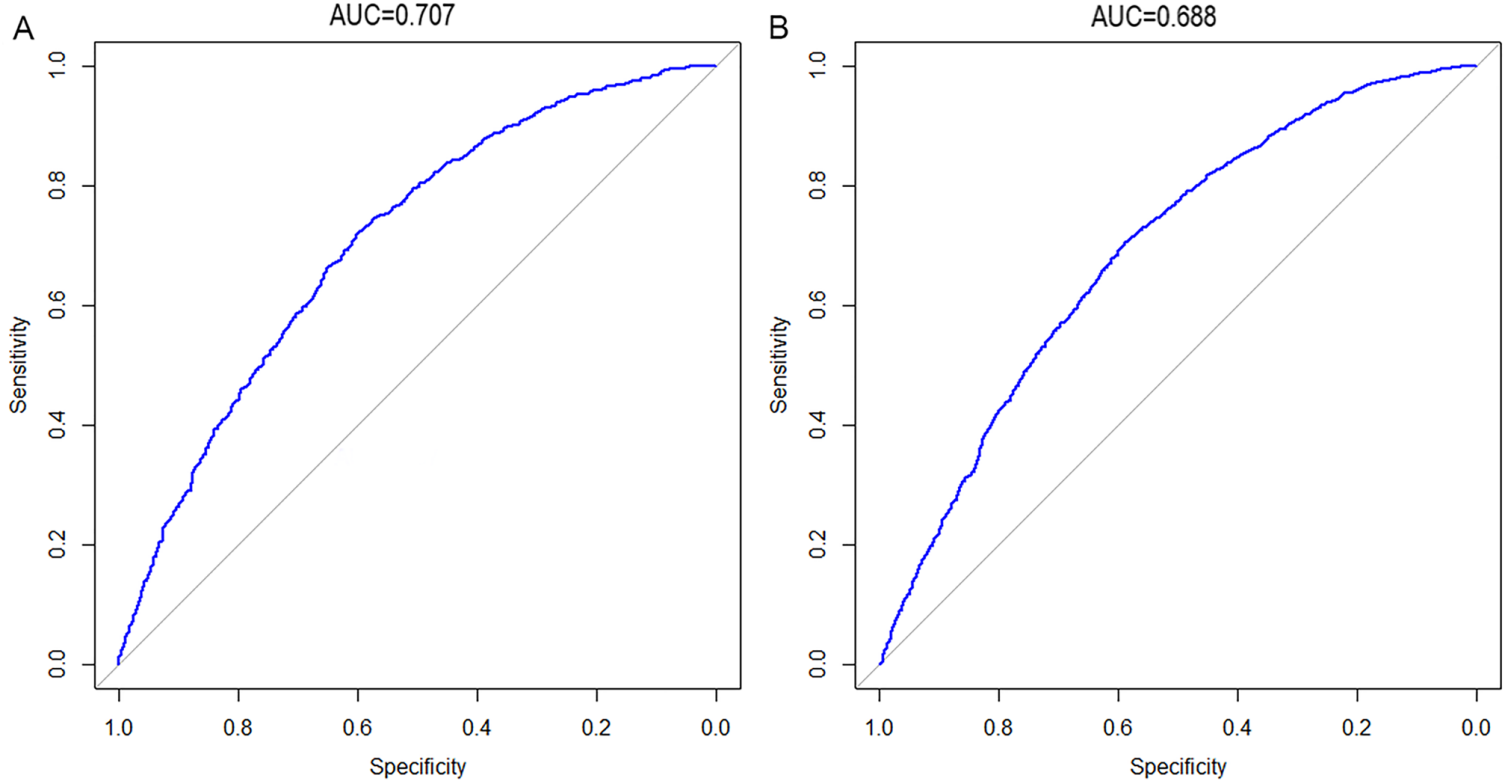
The area under the ROC curve is used to determine the predictive ability of the prediction model to distinguish outcomes, such as with or without disease. X-axis represents the false positive rate predicted by the model; Y-axis represents the true positive rate predicted by the model; the blue curve represents the performance of the nomogram. (**A**) The performance of the model in the training set. (**B**) The performance of the model in the validation set.

**Figure 5:**
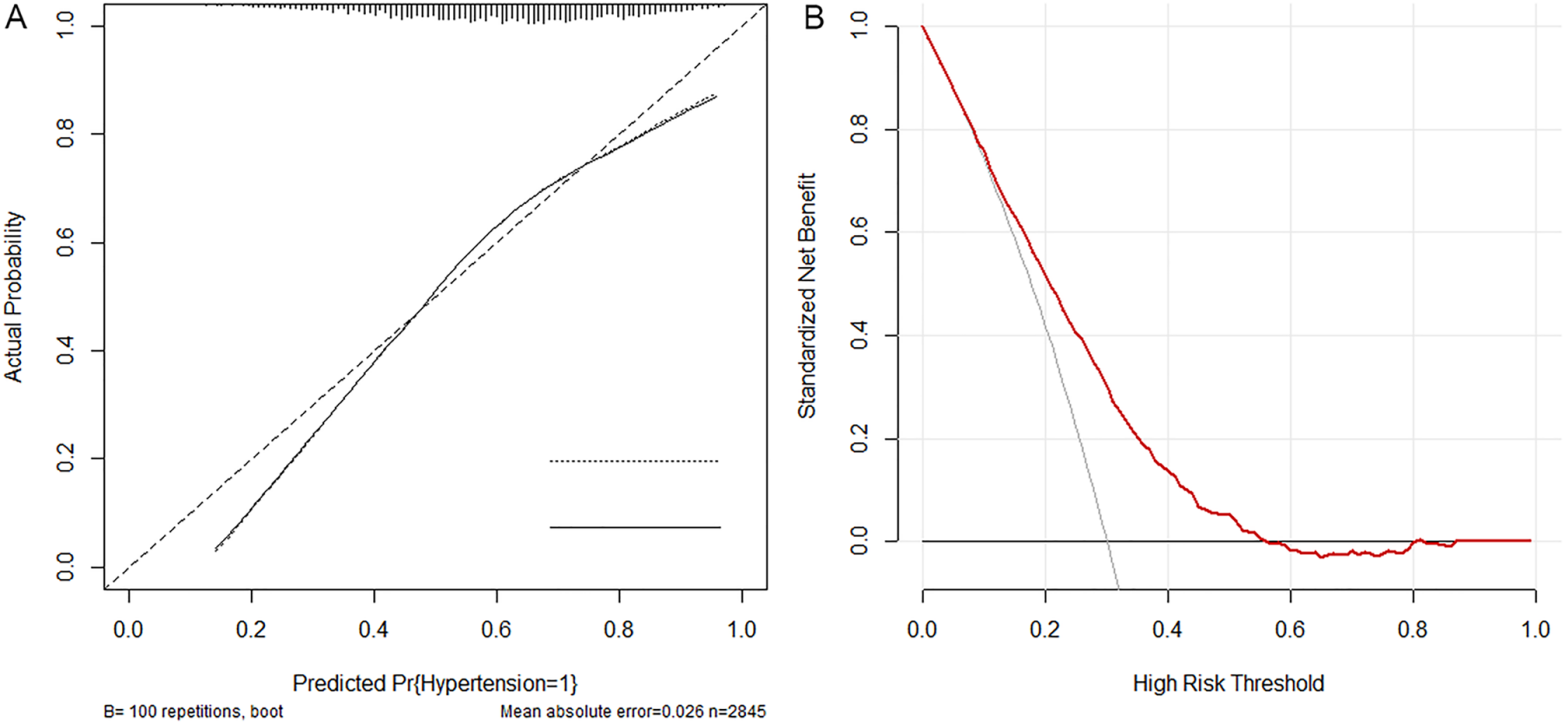
Analyses of the calibration and decision curves of the HBP risk nomogram. (**A**) The X-axis of the curve represents the predicted risk of concurrent Hypertension in elderly patients with NAFLD; Y-axis represents the actual diagnosed morbidity; the diagonal line represents the perfect prediction of the ideal model, and the higher overlap of the dark dashed line with the diagonal line represents the better accuracy of the model. (**B**) Decision curve analysis of the Hypertension risk nomogram. The solid line parallel to the horizontal axis in the graph indicates that when all samples are negative, the net benefit of having no intervention for all is 0. Backlash shows that all samples are positive and have received intervention, and the net benefit is the magnitude of the slope. The red line represents the net benefit of the model constructed in this study. The further the red line is from the two intersecting lines, the higher the clinical application value of the model.

**Figure 6:**
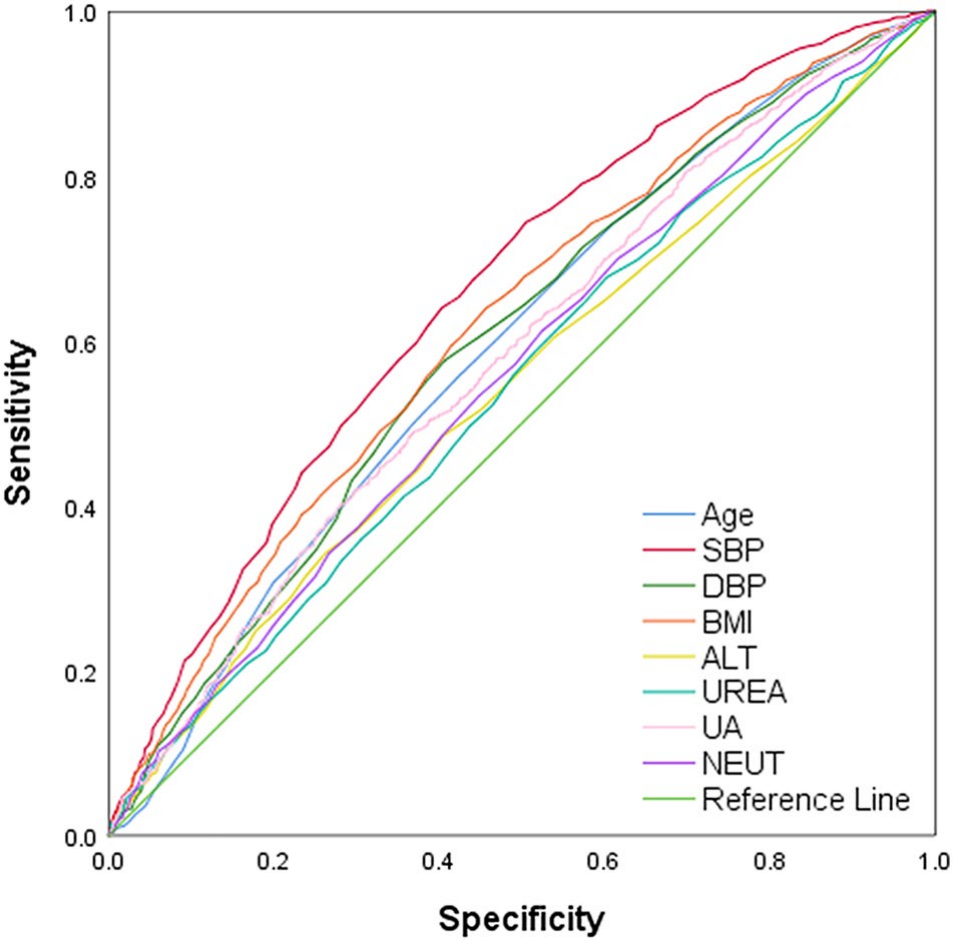
ROC curve analysis of the seven risk factors.

To evaluate the discrimination, calibration, and clinical applicability of the risk nomogram established in this study, the model was validated. The AUC of the internal validation set was 0.707 (95% CI: 0.688–0.727) and the AUC of the external validation set was 0.688 (95% CI: 0.672–0.705) (Fig. 4). The calibration curves showed moderate agreement. The results of the decision curves indicated that, selective patient treatment based on the results nomogram results in better patient outcomes than implementing an intervention-in-all-patients scheme, in cases where the risk threshold of Hypertension in NAFLD patients was 30–56% (Fig. 5). In addition, we analysed each variable in the model and plotted the ROC curve; the results are shown in Fig. 6, Table 3.

**Table 3:** The optimal cut-off value, specificity, and sensitivity of risk factors.

Characteristic	AUC	Cut-off value	Specificity (%)	Sensitivity (%)
Age	0.591	67.50	57.90	55.40
SBP	0.660	135.50	49.50	74.40
BMI	0.619	24.65	54.30	64.00
ALT	0.543	23.50	59.60	48.40
UREA	0.543	4.85	39.70	67.70
UA	0.580	360.15	63.00	49.10
NEUT	0.558	3.05	47.60	61.20

**Figure 7:**
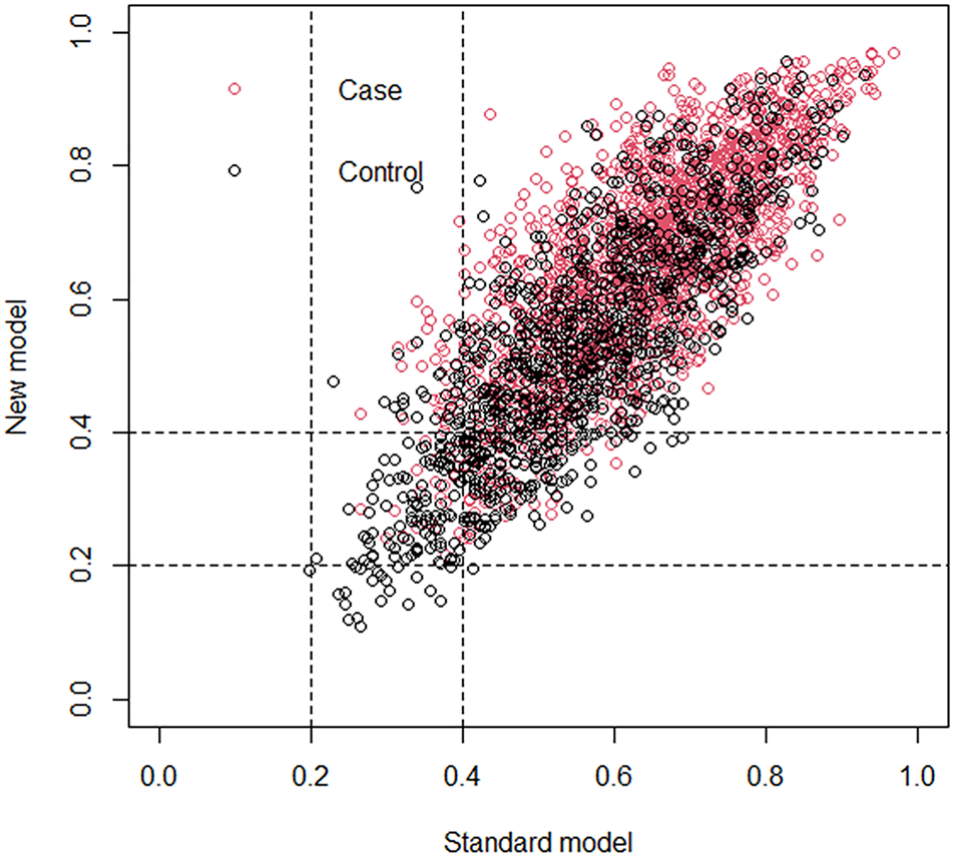
Model comparison based on NRI. The NRI value was 0.109.

Using only SBP and DBP (common indicators of Hypertension) model I was established in the NAFLD population (AUC was 0.660, 95% CI: 0.636–0.685). Model I was compared with the model constructed in this study using NRI; this produced a value of 0.109, indicating that the model constructed in this study had better predictive ability than model I (Fig. 7).

## Discussion

Based on the prediction model, we established that age, SBP, BMI, ALT, UREA, UA, and NEUT, are the risk factors for Hypertension development in patients with NAFLD. Among previous studies on predictive models for the risk of developing Hypertension in China, a longitudinal study by Zhang et al. analysed 26,496 Hypertension patients; they extracted 5 risk factors from 11 examined biomarkers to build a Hypertension Synthetic Predictor (HSP) for Hypertension, including Blood Pressure Factors (BPF) determined by SBP and DBP^23^. In a prospective study by Chen et al. that included 2,785 Hypertension patients, the variables of the established risk-prediction model were age, BMI, SBP, DBP, and fasting blood glucose (FBG)^24^. In addition, in a review study, 26 articles reporting on 48 Hypertension prediction models were analysed (seven domestic studies; others from Europe, the United States, Korea, Japan, and Iran). The most common variables in the Hypertension prediction models included age, BMI, and blood pressure level, among others^19^. Most studies on Hypertension prediction models in China and abroad included the predictive variables consistent with the results of this study.

Age is an irreversible risk factor in Hypertension development. Some studies have shown that Hypertension prevalence increases with age, and there is a strong correlation between biological ageing and elevated blood pressure^9,25–27^. SBP and DBP are common diagnostic indicators of Hypertension. This study also found that SBP was one of the main predictors of concurrent Hypertension and NAFLD, and that there is a correlation between BMI and Hypertension^28,29^. The results of a Hypertension prediction model study based on 135,715 individuals showed that a lower BMI reduced the risk of developing Hypertension^20^. Among patients with Hypertension in the United States, 49.5% are obese (BMI > 28)^30^. Moreover, there is strong evidence that obesity (including visceral obesity) is the most common risk factor for NAFLD^3,31^. The World Health Organization's BMI classification criteria are not suitable for application among Asians, and therefore we followed the classification system used in a previously published report, which presented the BMI classification for a Chinese population as follows: underweight (< 18.5), normal (18.5–22.9), overweight (23.0–27.5), and obese (> 27.5)^32^. According to this definition, the obesity rate of subjects with NAFLD and Hypertension in the present study was 73.8%.

Studies have shown that blood pressure is an important determinant of plasma UREA concentration, with a positive correlation between blood pressure and UREA concentration. In patients with untreated Hypertension, elevated blood pressure may be caused by renal insufficiency^33^. UREA abnormalities are also closely associated with kidney disease development^34,35^, suggesting that Hypertension is a cause or consequence of kidney disease^36^.

UA is associated with chronic kidney disease and cardiovascular disease. It can be a predictor of developing Hypertension in adults^37,38^ and is positively correlated with blood pressure. An increase in UA implies an increased risk of cardiovascular disease^39,40^. In a cross-sectional study of 33,570 patients with Hypertension by Ren et al., the established risk-prediction model included variables such as gender, age, height, weight, and UA^20^. Meanwhile, Rosa et al. proposed that UA is an important NAFLD predictor variable^41^.

The results of previous studies suggest that the development of cardiovascular diseases, including Hypertension, is closely associated with white blood cells (WBCs), a marker of inflammation^40,42,43^. An abnormal rise in WBC count may predict an increased risk of developing Hypertension. NEUT is a major component of WBCs and a correlation between NEUT and Hypertension has been noted in some studies, with higher NEUT values in patients with Hypertension^44^. In addition, some studies reported higher NEUT values in obese than in non-obese individuals^45,46^.

ALT is present in the liver and is the main marker of liver injury^47^. The population in our study was mainly composed of patients with NAFLD, so the predictor ALT is included. A few studies have explored the association between ALT and Hypertension development, with equally few studies demonstrating an association between ALT and Hypertension^48–50^. One of the cross-sectional studies conducted in Chinese adults suggested a positive linear correlation between ALT and Hypertension development.

Elevated blood pressure is strongly associated with increased dietary sodium intake^51–53^. In an article published in 2016, researchers conducted a dietary study across 20 Chinese provinces, which revealed that the per capita salt intake was 9.1 g/day and sodium intake was 5.4 g/day, both exceeding the recommended maximum daily salt intake (6 g/day) and sodium intake (2 g/day)^54^. Meanwhile, excessive intake of fats, sugars, and meat in the diet coupled with reduced physical activity are the main causes of increased BMI in the population^11^. A high-protein diet leads to excessive amino acids, which are oxidised into energy, in turn increasing UREA synthesis in the body^55^. To date, the basic treatment for excessive UA is dietary control and a study by Roumeliotis et al. suggested that increased intake of vitamins, such as vitamin C and E, can reduce UA levels^37^. The Dietary Approaches to Stop Hypertension program (DASH), a healthy dietary model proposed by the United States in 1997, emphasises that adequate intake of vegetables, fruits, and low-fat (or skimmed) milk is essential, while excessive consumption of animal fats should be avoided. Some studies have confirmed the effectiveness of this approach^11,56,57^. Based on the results of the present study, a low sodium, low sugar, high vitamin C and E, and moderate polyunsaturated fatty acid (PUFA) and protein diet should be selected for individuals with NAFLD and high Hypertension risk. An increase in moderate physical exercise is also recommended.

To date, interventions such as dietary control, physical exercise, and weight loss for patients with NAFLD remain some of the main basic therapies to delay or prevent the progression of the disease^58,59^. In addition, lifestyle changes play an important role in controlling blood pressure and preventing cardiovascular disease^60–62^ and are effective in controlling blood pressure in Hypertension patients and populations at high-risk of Hypertension. In particular, interventions such as a rational diet, weight control, smoking and alcohol consumption cessation, and stress reduction can be adopted in the early stages of Hypertension for high-risk groups^27,63,64^. Considering the commonalities between NAFLD and Hypertension management, our study primarily analysed the correlation between risk factors and certain dietary habits. We aimed to provide a reference for health management based on dietary regulation to help physicians formulate individualised dietary interventions for patients with NAFLD at high risk of developing Hypertension. We also aimed to raise patients' awareness of healthy dietary management through health education, adopt interactive follow-ups for timely access and provide feedback on the implementation of individualised dietary management and the real-time dynamics of key risk indicators.

## Conclusion

This study used the Lasso model based on tenfold cross-validation and an RF model to identify risk factors (age, SBP, BMI, ALT, UREA, UA, and NEUT) associated with NAFLD combined with Hypertension in the elderly population. Logistic regression models were used to obtain a simple, effective, and economical nomogram to predict the risk of comorbid Hypertension in NAFLD patients over the age of 60 in Shanghai, China.

Based on our results, for NAFLD patients with hypertension risk, we suggest that health service providers should strengthen the monitoring of seven risk indicators: age, SBP, BMI, alt, urea, UA, and NEUT. Health service providers can formulate and improve individualised intervention measures and risk monitoring systems to increase patients' quality of life and reduce the economic and medical burden imposed by NAFLD and Hypertension on the elderly, their families, and society.

This study has potential limitations. First, this is a retrospective study, and some clinical indicators with more vacancy values were eliminated, which may lead to unavoidable selection deviation. Such as some potential clinical indicators were not included. Second, this risk-prediction model has been established and validated in the Chinese Han population in Shanghai, but it lacks further study in other populations in other regions and countries. The model needs to be externally evaluated in a wider elderly population and needs further prospective research. The results of this study can therefore only be applied to the Chinese Han population; whether this model is applicable to the populations of other countries needs further verification. Third, our data cannot confirm whether individual intervention based on the predicted results of the model can reduce the risk of hypertension and improve the living standard of NAFLD patients; further large-scale and long-term follow-up studies are required to investigate this.

## Materials and methods

### Sample collection

**Figure 8:**
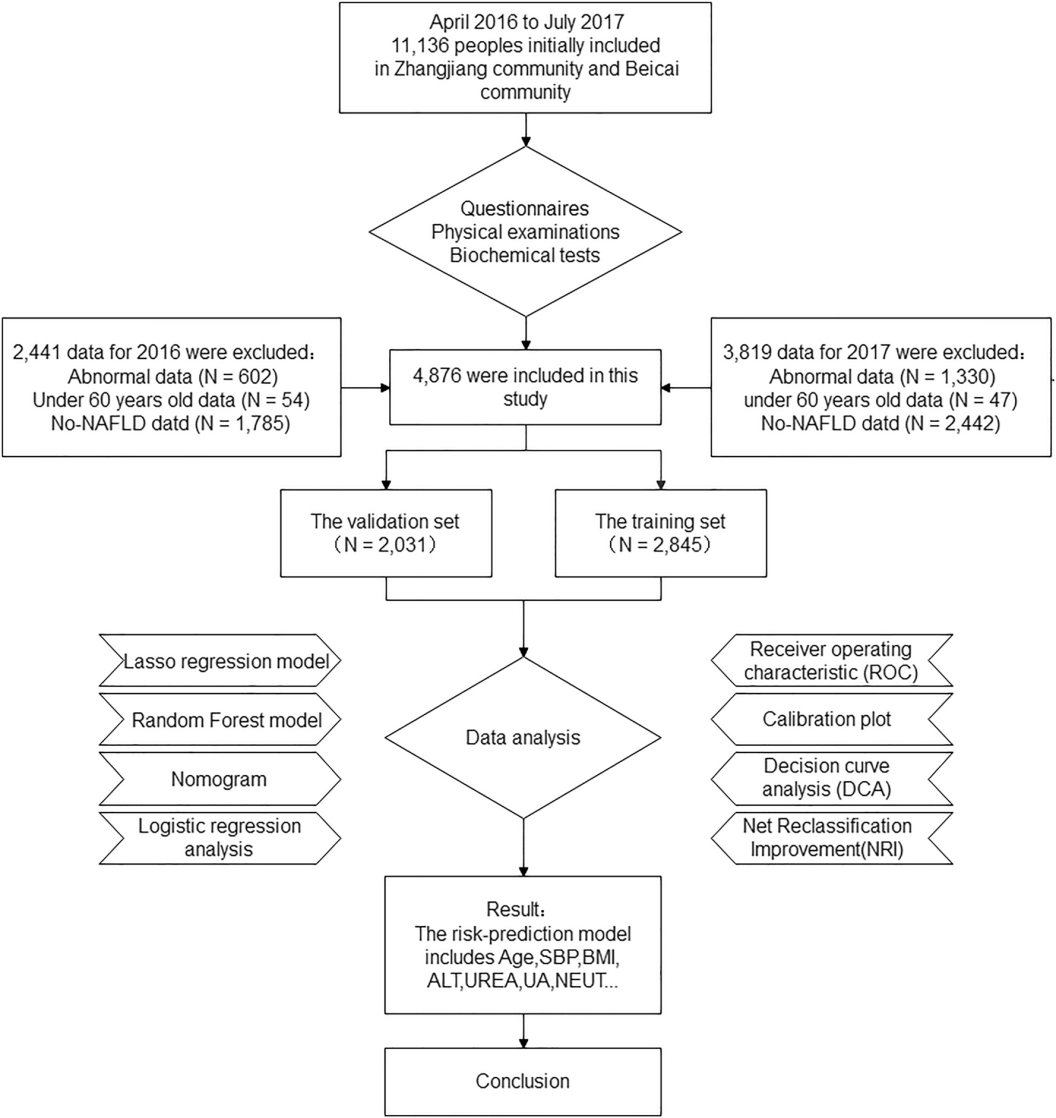
Flow diagram of the study design.

The sample data for this retrospective study were obtained from the Zhangjiang, Pudong New Area, Shanghai. We relied on the Shanghai Collaborative Innovation Center of Traditional Chinese Medicine Health Service of the Shanghai University of Traditional Chinese Medicine and the Zhangjiang and Beicai Community Health Service Centers, Pudong New Area, Shanghai, to collect the relevant information. This information was regarding the participants belonging to the Han population who participated in the chronic disease health screening programs conducted at the two community health centers from April 2016 to July 2017 using questionnaires, physical examinations, and biochemical tests. This study was conducted in accordance with the basic principles of the Declaration of Helsinki, and the protocol was approved by the Ethics Committee of the Shanghai University of Traditional Chinese Medicine. Informed consent was obtained from the study subjects and they signed an informed consent form (Fig. 8).

The research group collected 6,664 participants in 2017, who were matched for age and sex, randomised, and did not have blood relationship among the study subjects. After data processing, study subjects with missing information, incomplete indicators, and those over 60 years of age were excluded. The 5287 participants with who matched all criteria were divided into two groups: an NAFLD group (N = 2845) and a non-NAFLD group (N = 2442). The NAFLD group was the internal training set, and the external validation set comprised 2031 cases, which were obtained by pre-processing the data collected by the research group in 2016. The clinical characteristic indicators of the external validation set are consistent with those of the internal training set (see Supplementary Table S2 online).

The blood pressure of the subjects was measured using the steps recommended by international guidelines, including a quiet resting period of at least 5 min before measurement. An electronic sphygmomanometer (Biospace, Cheonan, Korea) was used to record each subject's SBP and DBP (mmHg).

The clinical diagnostic criteria in the "Guidelines for the diagnosis and management of nonalcoholic fatty liver disease (2010 Revision)" developed by the Chinese Society of Hepatology, CMA^65^, were used to determine the diagnostic criteria for NAFLD. The following three conditions had to be met:

1. No history of alcohol consumption. The quantity of ethanol consumption should be < 140 g/week for men and < 70 g/week for women.
2. Exclusion of other causes that could lead to hepatic steatosis, including viral hepatitis, drug-induced liver disease, autoimmune liver disease, hepatolenticular degeneration, and total parenteral nutrition.
3. Imaging or histological findings meet the diagnostic criteria of fatty liver disease.

Ultrasonography is used to identify hepatic steatosis. Ultrasonography evaluation of hepatic steatosis typically consists of a qualitative visual assessment of hepatic echogenicity, measurements of the difference between the liver and kidneys in echo amplitude, evaluation of echo penetration into the deep portion of the liver, and determination of the clarity of blood vessel structures in the liver. According to these imaging characteristics, liver steatosis is usually graded as mild (increased echogenicity but normal visualization of vessels and diaphragm), moderate (poor visualization of the intrahepatic vessels) and severe (diaphragm and deep parenchyma are not seen).

### Statistical and modelling methods

The analysis software used in this study were IBM SPSS Statistics (version: 25.0), Venny (version: 2.1.0), and R software (version: 4.1.0, https://www.R-project.org/), and the R packages were caret (version:6.0-88), DynNom (version:5.0.1), haven (version:2.4.3), readxl (version:1.3.1), rms (version:6.2-0), rmda (version:1.6), regplot (version:1.1), rsconnect (version:0.8.24), glmnet (version:4.1-2), nricens (version:1.6), pROC (version:1.17.0.1), and forestplot (version:2.1.0). The statistical tests used were all two-sided tests with test level α = 0.05.

The Lasso model based on tenfold cross-validation and RF model were used to screen the risk factors of Hypertension in patients with NAFLD. The Lasso model is suitable for variable screening of high-dimensional data, where the variables in the analysis are first pooled and normalised to reduce the regression coefficients of the variables toward zero, and the predictor variables with non-zero coefficients at the best lambda value are screened with the smallest possible prediction error^39,66^. The RF model is based on a recursive feature elimination approach that uses significant variables in the hierarchical ranking to train the random forest and eliminate irrelevant variables, followed by the repeated training and elimination processes until there is no variable change^67^.

In this study, the outcome variable was whether Hypertension occurred under NAFLD conditions. As it is a dichotomous variable, logistic regression analysis was used to construct a prediction model containing the final characteristic variables^18^. The Lasso model was intersected with the predictor variables screened using RF, and the identified common variables were included in the multivariate logistic regression analysis. The *p* > 0.05 predictor variables were gradually eliminated to obtain the final characteristic variables of the risk-prediction model, and the results were visualised using forest plots.

A nomogram can simplify and visualise complex regression equations, integrate data with a model, comprehensively consider the influential variables in the analysis, and graphically display the probability of the predicted outcome^68^. Therefore, we used the rms package in R software to plot the nomogram of the logistic regression model constructed in this study. The length of the line corresponding to each analysed variable in the plot indicated the degree of influence of that variable on the predicted outcome^69^.

### Model evaluation and comparison

We used the area under the ROC curve (AUC) to evaluate the discriminatory ability of the model in internal and external validation sets^70^. This value is generally between 0.5 and 1, and the closer to 1, the better the discriminatory ability of the model and the higher the predictive performance^21^. Appropriate calibration analysis is required when applying the prediction model constructed in this study to clinical decision-making^71^. To achieve this, we used the calibration curve to determine the degree of agreement between the actual status and the predicted results of the model. Because there is a possibility of false positives or false negatives in the prediction results of this model, we used DCA to determine the net benefit of the prediction model in clinical applications^18^. The NRI proposed in 2008 to evaluate the improvement of a new model in risk-prediction ability^72^ was applied in this study to compare the extent to which the newly constructed prediction model outperformed the prediction model constructed based on the traditional variables used in Hypertension diagnosis.

The flow chart of this study design is shown in Fig. 8.

## Supplementary Information

Supplementary Information.

## Data Availability

The datasets generated and analysed during the current study are available from the corresponding author on reasonable request.
