# Supplementary material for: Establishment and evaluation of a risk-prediction model for hypertension in elderly patients with NAFLD from a health management perspective
Source: Sci Rep. 2022 Sep 7;12:15138. doi: 10.1038/s41598-022-18718-3 (PMC9452675; doi:10.1038/s41598-022-18718-3)
Supplement: Supplementary file 1 — Supplementary Information. [file 41598_2022_18718_MOESM1_ESM.pdf]

Establishment and evaluation of a risk-prediction model for Hypertension in elderly patients with NAFLD from a health management perspective

An Zhang[1], Xin Luo[1], Hong Pan[1], Xinxin Shen[1], Baocheng Liu[2*], Dong Li[3*], Jijia Sun[2*]

[1] Department of health management, School of Public Health, Shanghai University of Traditional Chinese Medicine, Shanghai, 201203

[2] Shanghai Collaborative Innovation Center of Traditional Chinese Medicine Health Service, Shanghai University of Traditional Chinese Medicine, Shanghai, 201203

[3] Zhangjiang Community Health Service Centers, Pudong New Area, Shanghai, 201203

Supplementary Table S1 Differences in demographic and clinical characteristics between the lean and non-lean NAFLD groups in validation set.[mean ± SD or N(%)]

**Items**	**Lean (n=504)**	**overweight (n=1783)**	**obese (n=558)**	**χ2/F**	**P**
Hypertension					
Yes	221(43.8%)	1081(60.6%)	412(73.8%)	8.126	0.017
No	283(56.2%)	702(39.4%)	146(26.2%)		
Age	68.47±6.82	68.86±6.88	69.98±7.04	7.494	0.001
BMI	21.67±1.04	25.05±1.29	29.72±2.00	4376.480	< 0.001
SBP	138.68±21.20	144.67±20.86	149.72±21.19	36.671	< 0.001
DBP	79.58±11.33	82.17±11.32	84.98±10.87	30.756	< 0.001
WHR	0.87±0.06	0.90±0.06	0.92±0.06	94.624	< 0.001
ALB	44.61±2.63	44.62±2.54	44.17±2.36	6.851	0.001
ALT	23.57±12.43	26.05±13.67	27.89±15.71	12.926	< 0.001
AST	22.92±6.81	23.67±7.87	24.72±9.49	6.833	0.001
RBC	4.58±0.42	4.65±0.45	4.64±0.50	4.582	0.010
UREA	5.40±1.47	5.51±1.49	5.59±1.53	2.094	0.123*
GLU	6.36±2.12	6.36±1.75	6.45±1.62	0.548	0.578*
HGB	139.88±12.81	142.01±13.44	140.79±14.66	5.556	0.004
PLT	203.13±52.18	201.46±53.84	196.01±59.10	2.726	0.066*
TC	5.16±0.96	5.10±0.96	5.09±0.97	0.983	0.374*
TB	15.77±6.90	15.80±5.57	15.36±5.33	1.282	0.278*
CRE	66.18±16.22	68.98±17.62	70.40±19.18	7.878	< 0.001
LDL	3.22±0.86	3.20±0.85	3.26±0.87	0.872	0.418*
TG	1.64±1.12	1.74±1.31	1.69±0.90	1.537	0.215*
UA	343.19±84.96	360.96±89.64	365.53±81.31	10.258	< 0.001
AFP	6.77±5.34	6.80±4.42	7.26±4.77	2.228	0.108*
BASO	0.02±0.04	0.02±0.03	0.02±0.03	0.468	0.627*
EO	0.13±0.16	0.14±0.12	0.14±0.15	0.618	0.539*
LYMPH	1.87±0.53	1.95±0.59	2.00±0.61	7.034	0.001
NEUT	3.32±1.06	3.41±1.15	3.44±1.18	1.627	0.188*

note:"*" means P > 0.05 and the difference was not statistically significant

Supplementary Table S2 Differences in demographic and clinical characteristics between the No-NAFLD and NAFLD groups in validation set.[mean ± SD or N(%)]

Items	NAFLD (n=2031)	No-NAFLD (n=1785)	χ2/t	P
Hypertension				
Yes	1296(63.8%)	874(49.0%)	85.382	< 0.001
No	735(36.2%)	911(51.0%)		
Age	72.18±6.33	72.73±6.64	2.597	0.009
BMI	25.82±3.07	22.52±2.92	33.858	< 0.001
SBP	144.81±20.98	140.17±22.34	6.584	< 0.001
DBP	85.76±11.19	83.24±11.94	6.698	< 0.001
WHR	0.92±0.07	0.88±0.18	7.365	< 0.001
ALB	45.03±2.72	44.54±2.75	5.561	< 0.001
ALT	18.62±17.09	15.05±11.15	7.521	< 0.001
AST	20.15±9.00	19.62±7.31	2.008	0.045
RBC	4.55±0.44	4.43±0.45	8.440	< 0.001
UREA	5.69±1.46	5.85±1.58	3.297	0.001
GLU	5.47±1.85	5.74±1.92	4.548	< 0.001
HGB	137.52±13.03	133.80±13.69	8.575	< 0.001
PLT	199.45±48.06	194.82±47.28	2.991	0.003
TC	4.88±0.91	4.74±0.93	4.718	< 0.001
TB	11.10±4.55	11.02±5.54	0.540	0.589*
CRE	76.01±19.92	76.75±19.62	1.142	0.254*
LDL	3.12±0.80	3.02±0.80	4.200	< 0.001
TG	1.62±1.07	1.15±0.73	15.779	< 0.001
UA	314.75±84.68	290.37±81.44	9.034	< 0.001
AFP	6.54±3.78	5.42±3.97	1.247	0.213*
BASO	0.03±0.02	0.03±0.02	0.031	0.975*
EO	0.13±0.12	0.13±0.12	0.992	0.321*
LYMPH	2.10±0.76	1.92±0.60	8.277	< 0.001
NEUT	3.65±1.14	3.47±1.17	4.844	< 0.001

note:"*" means P > 0.05 and the difference was not statistically significant
